# Gender Roles and Physical Function in Older Adults: Cross-Sectional Analysis of the International Mobility in Aging Study (IMIAS)

**DOI:** 10.1371/journal.pone.0156828

**Published:** 2016-06-03

**Authors:** Tamer Ahmed, Afshin Vafaei, Mohammad Auais, Jack Guralnik, Maria Victoria Zunzunegui

**Affiliations:** 1 Département de médecine sociale et préventive, Université de Montréal, Montréal, QC, Canada; 2 Department of Public Health Sciences, Queen's University, Kingston, ON, Canada; 3 Department of Epidemiology and Public Health, Division of Gerontology, University of Maryland School of Medicine, Baltimore, Maryland, United States of America; Nathan Kline Institute and New York University School of Medicine, UNITED STATES

## Abstract

**Objectives:**

To examine the relationships between physical function and gender-stereotyped traits and whether these relationships are modified by sex or social context.

**Methods:**

A total of 1995 community-dwelling older adults from the International Mobility in Aging Study (IMIAS) aged 65 to 74 years were recruited in Natal (Brazil), Manizales (Colombia), Tirana (Albania), Kingston (Ontario, Canada), and Saint-Hyacinthe (Quebec, Canada). We performed a cross-sectional analysis. Study outcomes were mobility disability, defined as having difficulty in walking 400 meters without assistance or climbing a flight of stairs without resting, and low physical performance, defined as a score < 8 on the Short Physical Performance Battery. The 12-item Bem Sex Role Inventory (BSRI) was used to classify participants into four gender roles (Masculine, Feminine, Androgynous, and Undifferentiated) using site-specific medians of femininity and masculinity as cut-off points. Poisson regression models were used to estimate prevalence rate ratios (PRR) of mobility disability and poor physical performance according to gender roles.

**Results:**

In models adjusted for sex, marital status, education, income, and research site, when comparing to the androgynous role, we found higher prevalence of mobility disability and poor physical performance among participants endorsing the feminine role (PRR = 1.20, 95% confidence interval (CI) 1.03–1.39 and PRR = 1.37, CI 1.01–1.88, respectively) or the undifferentiated role (PRR = 1.23, 95% CI 1.07–1.42 and PRR = 1.58, CI 1.18–2.12, respectively). Participants classified as masculine did not differ from androgynous participants in prevalence rates of mobility disability or low physical performance. None of the multiplicative interactions by sex and research site were significant.

**Conclusion:**

Feminine and undifferentiated gender roles are independent risk factors for mobility disability and low physical performance in older adults. Longitudinal research is needed to assess the mediation pathways through which gender-stereotyped traits influence functional limitations and to investigate the longitudinal nature of these relationships.

## Introduction

Mobility is fundamental for autonomy, independence, and high quality of life [1–3]. It is associated with time spent outside the home and overall health perceptions among older adults [4]. As population ageing is a notable phenomenon in both developed and developing countries, mobility loss will remain a significant public health challenge [5] for the foreseeable future, as it is a risk factor for falling, disability, hospitalization, long-term health care costs, and mortality.

Gender refers to the array of socially constructed roles and relationships, personality traits, attitudes, behaviours, values, relative power, and influence that society ascribes to men and women on a differential basis [6, 7]. Sex is a biological construct that refers to the biological differences between females and males and is distinct from, and not interchangeable with, gender [8]. Gender differences in mobility disability among older adults have been observed in numerous studies, but are not well understood. Most studies focused on understanding the biological differences in mobility disability between men and women, but not the differences due to interrelationships of sex and gender. Many studies have demonstrated that women have greater prevalence and incidence of mobility disability than men [9–13]. Interestingly, the magnitude of difference in mobility disability between older men and women varies across studies and locations worldwide [14].

In the few studies conducted in low and middle income countries, a larger gap in mobility disability between men and women has been observed [15–17]. Baseline analysis of data from the International Study of Mobility in Aging (IMIAS) on older adults between 65 and 74 years at five research sites in Natal (Brazil), Manizales (Colombia), Tirana (Albania), Saint-Hyacinthe (Quebec), and Kingston (Ontario) suggests that women had a statistically significant higher odds of mobility disability (defined as having difficulty in walking 400 meters or climbing a flight of stairs) at all research sites except Kingston (Ontario) [18].

Several reasons for these gender differences have been reported in the literature. Women generally live longer with greater functional limitations [13], which means they spend more years at risk of disabilities than men [19, 20], resulting in higher prevalence. It has been hypothesized that the greater prevalence among older women of osteoarthritis and musculoskeletal diseases, with their associated pain [21, 22] and depression [23], may partly explain gender differences in mobility disability.

Given the widespread use of conventional self-report tools for mobility assessment, differential reporting of mobility difficulty by men and women could contribute to the observed sex-based differences in mobility. These self-assessments can be improved by adding objective physical performance measures [13, 24, 25]. Lower physical performance has been observed in women who are younger at first birth and multiparous, two frequent factors among women living in poor and middle income countries [26] that could explain the varying mobility gap between older men and women. Differences in mobility between men and women have been also explained by gender roles that vary across times periods and world regions [14, 17, 27]. Differences in the social construction of gender stem from social factors and behavioural responses that depend on time and place. Social factors are expressed through norms and values imposed by a number of societal and cultural institutions, such as government laws, family roles and traditions, religion, and mass media, while behavioral responses are the expression of social norms and values through individual attitudes and behaviours. Women in some societies do not enjoy equal rights and are more often exposed to violence, discrimination and stigmatization compared to men. Such adverse living conditions may lead to physiological mechanisms and ultimately contribute to gender differences in mobility disability in early old age.

Over the past few years, the concept of gender role orientation (GRO) has been introduced as a psychological measure for studying gender differences in health. According to the androgyny model, individuals who endorse high masculinity and femininity are classified as ‘androgynous’, those high in masculinity and low in femininity as ‘masculine’, those high in femininity and low in masculinity as ‘feminine’, and those low in both as ‘undifferentiated’ [28]. The association between biological sex and gender role orientation is not consistent across previous studies reported in the literature. For instance, results from studies conducted among young German nurses do not support sex differences regarding gender roles [29]. In two pilot studies conducted by our team in Brazil and Spain, gender roles were not statistically associated with biological sex in older adults [30, 31]. It is worth noting that the sample sizes of all of the aforementioned studies were small. Meanwhile, findings from the oldest cohort of the longitudinal study of social patterning of health from Scotland showed small but significant sex differences regarding gender roles (p<0.05) [32].

GRO, along with the Bem Sex Role (BSRI) classification system, has been little used in health-related research. Empirical research findings on GRO and health suggest that, among men, ‘masculinity’ contributes to higher risk of chronic heart disease (CHD) mortality, while higher ‘femininity’ scores are associated with a lower risk of CHD death [32]. Among men and women from the same study, ‘masculinity’ was negatively related to suicidal thoughts in early middle age, while ‘femininity’ was unrelated to serious suicidal thoughts at any age [33]. A small study of healthy middle-aged workers from Montreal, Canada, revealed that higher masculinity and female sex predicted increased physical complaints, with findings suggesting increased vulnerability to cardiovascular diseases [34]. Higher femininity was associated with higher rates of recurrent acute coronary syndrome (ACS) and, among younger patients with ACS, with increased risk of hypertension, a family history of cardiovascular diseases, and depressive and anxious symptoms [35, 36]. Androgynous gender roles [37] and higher masculinity scores [38] were associated with lower depressive symptoms in older adults.

Pain has been also linked to gender roles. A meta-analysis of thirteen studies showed masculinity was associated with higher pain threshold and pain tolerance, while femininity was associated with greater pain sensitivity response in healthy human participants. However, these results should be interpreted with caution given remarkable heterogeneity between studies [39]. Among older adults, femininity was associated with greater pain perception in men and lower pain sensitivity in women [40].

Health services use is generally associated with being a woman. However, one study showed that both older men and women with increased masculinity visit health services more often [41]. Findings of a study among Tokyo metropolitan centenarians suggest that femininity is related to longevity and that androgyny may be related to successful aging [42].

None of the studies cited has examined the associations between gender roles and physical function in old age. The unusual variability of the sex gap in mobility disability worldwide has raised the question of whether mobility loss is related to femininity while good physical function is related to masculinity. Therefore, we developed the following hypotheses:

1. Gender roles are associated with mobility disability and physical performance of the lower extremities in old age.
2. Compared to androgynous types, men and women who identify themselves with characteristics identified as ‘masculine’ have lower prevalence ratios of both mobility disability and low physical performance, while those identified as ‘undifferentiated’ or ‘feminine’ have higher prevalence ratios.
3. The strength of associations between gender roles, mobility disability, and physical performance varies across societies.

## Methods

### Population and samples

#### Sampling and recruitment

Participants are from the baseline survey of the International Mobility in Aging Study (IMIAS). The aim of the study is to examine gender differences in mobility using a life course perspective. The IMIAS was conducted in five cities located in countries with different degrees of gender equity to provide a wide range of gender-related exposure, mobility risk factors, and physical function outcomes. The rationale for the study has been explained in previous publications (Zunzunegui, 2015). Briefly, the study took place in two Canadian cities (Kingston, Ontario, and Saint-Hyacinthe, Quebec), one city in the Colombian Andes (Manizales), one city in northeastern Brazil, and Tirana in Albania. The majority of older adult populations in Brazil, Albania, and Canada are registered in national public health systems that provide universal health insurance coverage. In Colombia, approximately 82% of adults over 60 years of age are covered under social security systems and subsidized public health programs [43].

A total of 1995 community-dwelling elderly people aged between 65 and 74 years were recruited in 2012. The sample was stratified by sex, consisting of approximately 200 men and 200 women from five research sites: Natal (Brazil), Manizales (Colombia), Tirana (Albania), Kingston (Ontario, Canada), and Saint-Hyacinthe (Quebec, Canada). Sample size calculations were performed assuming a baseline mobility disability prevalence ratio between men and women of 1.8, with a type I error of 0.05 and type II error of 0.2.

#### Recruitment strategies

Recruitment has been described in previously published reports [18, 44]. Briefly, two recruitment methods were used. In Natal, Tirana, and Manizales participants were selected randomly through their neighborhood primary care centers, and interviewers contacted participants directly to invite them to participate in the study.

Participants from Canadian sites were recruited randomly, with replacement, from neighborhood family medicine clinics. Family doctors sent invitation letters to their patients to contact our field coordinator for information about the study. In Saint-Hyacinthe, all patients came from the largest family medicine group clinic, with which more than 80% of physicians are affiliated and which covers the whole territory of the city. In Kingston, the two clinics included in the study are large, covering the whole Central Kingston area. In Saint-Hyacinthe the sample was stratified by neighbourhood, which increased the representativeness of the study sample, while in Kingston such stratification was not possible. As a result, the Saint-Hyacinthe sample was representative of the community’s older adult populations aged between 65 and 75 years in terms of marital status, education and income, as compared to the 2006 Canadian census. In Kingston, participants were more educated than the general census population of that site, but had similar marital status and income.

These indirect methods of recruitment in the Canadian cities were necessary because the ethics committees for the Canadian sites did not authorize researchers to communicate directly with potential participants to invite them to participate.

While the response rate was close to 100% at the Latin American sites and greater than 90% at the Albanian site, the response rate at Canadian sites was approximately 30%. Existing literature suggests that response rates can be expected to be lower at Canadian sites than at other participating research sites [45–47], mostly due to restrictions on direct access to participants.

#### Data collection methods

Responses to the IMIAS questionnaire were gathered through detailed structured interviews that included a wide range of measures of demographic and socioeconomic variables, self-report of existing medical conditions, life space assessment, health behaviours, quality of life, physical activity, physical development and tests of cognitive function, as well as an assessment of grip strength, vision, and blood pressure. All interviewers received standardized training at each site. All data collection procedures were carried out at the participants’ homes, except in Manizales, where vision and physical performance tests were carried out at the local hospital. All procedures, including data collection documents and manuals, are available in local languages.

#### Exclusion criteria

Participants were excluded if they had four or more errors on the Orientation Scale of the Leganes Cognitive Test (LGT) [48], administered at the beginning of the interview, since in that case they were considered to be incapable of completing the study procedures. In all, nine participants were excluded for this reason: five in Natal, two in Manizales, one each in Saint-Hyacinthe and Tirana, and none in Kingston.

#### Ethical considerations

The IMIAS study was approved by the research ethics committees of the University of Caldas (Colombia), the Universidad Federal do Rio Grande do Norte (Brazil), the Albanian Institute of Public Health (Albania), Queens University (Canada), and the University of Montreal Hospital Research Centre (Canada). Written informed consent was obtained from all subjects before their participation.

### Outcomes

This study has two outcomes: mobility disability and poor physical performance. Mobility disability is self-reported difficulty in walking 400 meters or climbing a flight of stairs without resting [49].

Physical performance was assessed using a battery of tests of lower extremity function, the Short Physical Performance Battery (SPPB). This objective measure is a strong predictor of mobility loss in older adults; individuals with low scores are more likely to suffer disability, hospitalizations, and mortality [50–52]. SPPB includes three timed tests of lower extremity function: a hierarchical test of standing balance, a four-meter walk, and five repetitive chair stands. For the standing balance test, participants were instructed to maintain a bipedal stance for 10 seconds, followed by a semi-tandem stance for 10 seconds. The gait speed task involved timing a four-meter walk at the participants’ normal pace. For those without a four-meter course in their home, a three-meter test was conducted and scoring adjusted accordingly. This test was repeated twice, with the faster of the two walks used. For the chair standing task, participants were first asked to demonstrate their ability to rise once from a chair. If they demonstrated this ability, they were asked to stand up and sit down five times as quickly as possible with their arms folded across their chests. Further details on administering these three tests have been published elsewhere [53, 54] and can be viewed on the SPPB website (http://www.grc.nia.nih.gov/branches/leps/sppb/index.htm). Each of the three SPPB components (balance, gait, and chair stands) is scored from 0 to 4, with 0 indicating inability to perform the test, and 4 indicating the highest category of performance. A summary of participants’ physical performance scores was obtained by adding up the scores of all three SPPB components for each participant. Total scores could thus vary from 0 to 12, with higher scores representing better physical performance.

We have validated SPPB in French, Spanish, and Portuguese during previous studies conducted in Quebec, Colombia, and Brazil [55, 56]. For this study, poor physical performance was defined as a total SPPB score below 8 [18].

### Exposure

#### Gender role orientation

Gender roles were measured using the 12-item Short Form of the Bem Sex Role Inventory (BSRI), covering stereotyped traits. This tool was originally developed and tested among a sample of university students from Spain [57]. The validity of the 12-item BSRI has been demonstrated with Spanish and Brazilian older adults [30, 31]. In confirmatory factor analysis using IMIAS data, two-factor solutions were extracted from the 12-item BSRI, and the final model has demonstrated acceptable convergent and construct validity for measuring gender roles in older adults [58].

The ‘masculinity’ and ‘femininity’ scores are each the **mean of six item ratings** (**‘masculinity or instrumentality’**–has leadership abilities, acts like a leader, is dominant, has strong personality, defends own beliefs, makes decisions easily; **‘femininity or expressiveness’**- tender, warm, affectionate, gentle, sympathetic, sensitive to others needs) (Table 1).

**Table 1:** Distribution of Masculinity and femininity scores in IMIAS sample (n = 1967).

	Masculinity scores^a^	Femininity scores^a^
	mean (SD) median	mean (SD) median
	Entire sample (n = 1967)	Entire sample (n = 1967)
**Study site**		
Kingston (n = 393)	5.14 (0.96) 5.17	5.62 (0.85) 5.67
Saint-Hyacinthe (n = 392)	4.75 (1.14) 4.83	5.74 (0.75) 5.83
Tirana (n = 387)	4.74 (1.12) 4.83	6.15 (0.74) 6.33
Manizales (n = 393)	4.22 (1.14) 4.17	5.92 (1.08) 6.33
Natal (n = 402)	4.28 (1.30) 4.17	5.32 (0.99) 5.33
	P <0.001^b^	P <0.001^b^

^**a**^ Masculinity and femininity scores were calculated as means of ratings of the six items in each scale.

^**b**^ One-way ANOVA for equality of means by study site.

The internal reliability of both scales was acceptable, with Cronbach’s α = 0.75, and α = 0.76 for instrumentality and expressiveness items respectively and for men and women separately (α ranged between 0.73 and 0.78) [58]. BSRI questions were administered to participants using visual aids illustrating a scale from 1 (‘never or almost never true’) to 7 (‘always or almost always true’) for each item. The median-split method recommended by Bem was used to dichotomize ‘masculinity’ and ‘femininity’ scores; thus, scores greater than or equal to the median score were classified as ‘high’, those with a score below the median as ‘low’. This resulted in four distinct groups, depending on whether individuals scored higher or lower than the median on the ‘masculinity’ and ‘femininity’ scales: ‘androgynous’ [high ‘femininity’ score (F), high ‘masculinity’ score (M)]; ‘masculine’ [high M, low F]; ‘feminine’ [high F, low M]; or ‘undifferentiated’ [low M, low F] [28]. We used the median value of each IMIAS research site to account for site-specific differences.

### Covariates

Age, sex, marital status, education, income, and study site were considered potential confounders for the association between gender roles and mobility disability in older adults. As people live longer, they are more likely to develop mobility limitations and disability [59–61]. Since the IMIAS population ranges in age from 65 to 74 years, those who were older had greater life experience and belonged to earlier cohorts in their respective countries, which may have affected their personal views of gender role orientation.

Participants were asked if they were single, married, widowed, or divorced. Little is known about the association between gender roles and marital status, but there is a possible link between them. For instance, married men from Japan tended to have higher masculinity than unmarried men [62].

Socioeconomic status is associated with gender roles. Men and women with higher education levels and income endorse more egalitarian gender role orientations [63, 64]. Meanwhile, those endorsing traditional gender roles are characterized by low socioeconomic status (low income and education levels) [65, 66]. We used education as a continuous variable to eliminate residual confounding, since the mean years of education varied across sites. Sufficiency of income was self-reported using the following question: ‘To what extent is your income sufficient to meet your ends?’ The possible responses were very sufficient, sufficient, and insufficient.

Since IMIAS is carried out in five research sites with different cultural, political and economic backgrounds, adjustments were made by research site.

### Statistical analysis

Data analysis was performed with the IBM Statistical Package for Social Sciences (SPSS) version 22.0 and with STATA version 11.0. We restricted our analyses to participants for whom no data was missing data for any of the BSRI items or any of the covariates. Participants with missing values on any of the BSRI items (n = 28) were not different from those included in data analyses in terms of age, sex, years of education, occupation type, income sufficiency, or research site (p>0.05). The final sample consisted of 1967 participants across the five IMIAS research sites. Descriptive statistics were obtained for the total sample by sex. Differences between gender role groups and other covariates by sex were examined statistically using Chi-square tests, T-tests, and ANOVA tests where appropriate.

Bivariate statistics were used to investigate and assess the relationships between gender role groups and the co-variates. A series of Poisson regression models with robust variance was run for the whole IMIAS sample. Poisson regression is preferred over logistic regression when analyzing common binary outcomes in cross-sectional data, because it provides estimation of the prevalence rate ratios with more conservative confidence intervals [67]. These models assessed the relationship between gender roles and self-reported mobility disability or low physical performance, using the androgynous role as the reference category and adjusting for potential covariates. Multiplicative product terms were added to test for interactions between a) gender role groups and biological sex b) gender roles and research sites, at a statistical significance level of p<0.05.

## Results

All of the 1967 participants (1025 women and 942 men) were born between 1938 and 1947, and the mean age for men (69.13, 2.92 SD) and women (69.10, 2.80 SD) was approximately similar (Table 2).

**Table 2:** Distribution of IMIAS participants according to gender roles and covariates by sex.

	Men			Women			
	(n = 942)			(n = 1025)			
	n	%	Mean (SD)	n	%	Mean (SD)	p-value
**Masculinity score**	942		4.78 (1.15)	1025		4.49 (1.21)	<0.001
**Femininity score**	942		5.60 (0.97)	1025		5.88 (0.88)	<0.001
**Median split method**							<0.001
‘Feminine’	136	14.4		277	27.0		
‘Masculine’	245	26.0		153	14.9		
‘Undifferentiated’	267	28.3		260	25.4		
‘Androgynous’	294	31.2		335	32.7		
**Age**	942		69.13 (2.92)	1025		69.10 (2.80)	0.79
**Years of education**	942		10.36 (6.07)	1025		9.06 (5.43)	<0.001
**Marital status**							<0.001
Single	41	4.4		79	7.7		
Married	754	80.0		517	50.7		
Widowed	44	4.7		281	27.4		
Divorced	103	10.9		148	14.4		
**Income sufficiency**							0.01
Very sufficient	312	33.1		284	27.7		
Barely sufficient	317	33.7		345	33.7		
Insufficient	313	33.2		396	38.6		
**Study site**							0.95
Kingston	184	19.5		209	20.4		
Saint-Hyacinthe	188	20.0		204	19.9		
Natal	192	20.4		210	20.5		
Manizales	195	20.7		198	19.3		
Tirana	183	19.4		204	19.9		

The distribution of men and women was not different across research sites (p = 0.85). Mean years of education was slightly higher for men (10.36, 2.92 SD) than for women (9.06, 5.43 SD). Marital status of participants varied significantly by sex, with fewer men than women reported as single (4.4% vs.7.7%), more men than women married (80% vs. 50.7%), fewer men than women widowed (4.7% vs. 27.4%), and fewer men than women divorced (10.9% vs. 14.4%). Income sufficiency varied significantly by sex: more men than women reported very sufficient income (33.1% vs. 27.7%), equal proportions of men and women reported barely sufficient income (33.7%), and fewer men than women reported insufficient income (33.2% vs. 38.6%). Men reported higher scores on the masculinity scale than women (mean 4.78 vs. 4.49, p<0.001) and lower scores on the femininity scale than women (mean 5.60 vs. 5.88, p<0.001).

**Fig 1:**
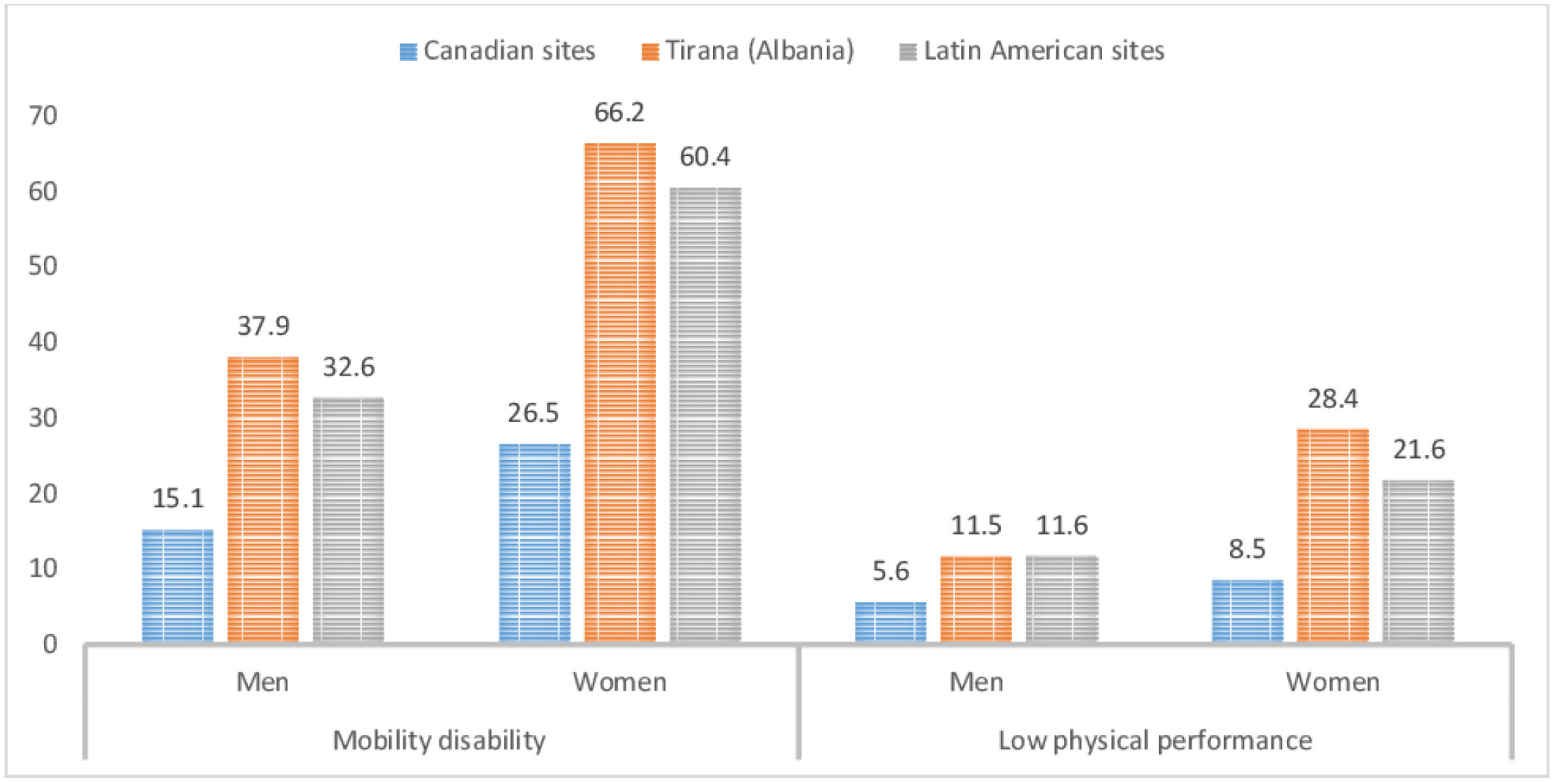
Sex-specific prevalence of mobility disability and low physical performance (%) across different research sites of IMIAS.

Gender roles varied significantly by biological sex (p<0.001). More men than women were classified as masculine (26% vs. 14.9%) and undifferentiated (28.3% vs 25.4%), and fewer as feminine (14.4% vs 27%). Approximately similar proportions of men and women were classified as androgynous (31.2% vs 32.7%). At all IMIAS research sites, women reported higher prevalence of mobility disability and low physical performance, with the highest prevalence in Tirana, and the lowest at Canadian sites (p<0.001) (Fig 1).

Table 3 shows the sex-specific relationships between gender role groups, functional limitations, and all potential confounders. In men, low physical performance was more frequent for the undifferentiated gender role; having very sufficient income was more frequent among the androgynous and masculine types. In women, mobility disability and poor physical performance were more frequent in the undifferentiated type; years of education were highest in the androgynous and lowest in the undifferentiated types; and having very sufficient income was more frequent among androgynous women and less frequent among those in the undifferentiated type.

**Table 3:** Relationship between BSRI 4 –fold classification, mobility disability and low physical performance, and covariates by sex.

	Men (n = 942)									Women (n = 1025)								
	Feminine		Masculine		Undifferentiated		Androgynous		P value	Feminine		Masculine		Undifferentiated		Androgynous		P value
	n	%	n	%	n	%	n	%		n	%	n	%	n	%	n	%	
**Mobility disability** ^a^																		0.002
**Difficulty**	41	30.1	64	26.1	80	30.2	66	22.4	0.156	137	49.5	72	47.4	146	56.2	135	40.4	
**No difficulty**	95	69.9	181	73.9	185	69.8	228	77.6		140	50.5	80	52.6	114	43.8	199	59.6	
**Physical performance SPPB** ^a^									0.04									0.006
**< 8**	10	7.4	20	8.3	36	13.7	21	7.3		55	20.3	23	15.1	59	22.7	42	12.6	
**≥ 8**	126	92.6	220	91.7	226	86.3	267	92.7		216	79.7	129	84.9	201	78.7	291	78.4	
**Age** ^b^	136	69.24	245	69.17	267	69.07	294	69.12	0.952	277	69.26	153	69.07	260	68.99	335	69.07	0.702
		(2.97)		(2.89)		(2.88)		(2.97)			(2.84)		(2.89)		(2.69)		(2.83)	
**Marital status** ^a^									0.152									0.33
**Single**	6	4.4	8	3.3	17	6.4	10	3.4		19	6.9	15	9.8	24	9.2	21	6.3	
**Married**	103	75.7	201	82.0	220	82.4	230	78.2		142	51.3	82	53.6	124	47.7	169	50.4	
**Widowed**	8	5.9	14	5.7	6	2.2	16	5.4		84	30.3	38	24.8	65	25.0	94	28.1	
**Divorced**	19	14.0	22	9.0	24	9.0	38	12.9		32	11.6	18	11.8	47	18.1	51	15.2	
**Years of education** ^b^	136	10.13	245	10.42	267	9.79	294	10.93	0.158	277	8.90	153	9.11	260	8.38	335	9.70	0.029
		(5.82)		(6.17)		(6.20)		(5.96)			(5.00)		(5.67)		(5.46)		(5.59)	
**Income sufficiency** ^a^									0.014									0.002
**Very sufficient**	35	25.7	98	40.0	75	28.1	104	35.4		81	29.2	40	26.1	52	20.0	111	33.1	
**Barely sufficient**	56	41.2	65	26.5	101	37.8	95	32.3		99	35.7	42	27.5	90	34.6	114	34.0	
**Insufficient**	45	33.1	82	33.5	91	34.1	95	32.3		97	35	71	46.4	118	45.4	110	32.8	
**Study site** ^a^									0.065									0.203
**Kingston**	26	19.1	38	15.5	63	23.6	57	19.4		54	19.5	29	19.0	43	16.5	83	24.8	
**Saint-Hyacinthe**	32	23.5	48	19.6	42	15.7	66	22.4		59	21.3	34	22.2	55	21.2	56	16.7	
**Natal**	27	19.9	42	17.1	52	19.5	71	24.1		46	16.6	32	20.9	60	23.1	72	21.5	
**Manizales**	28	20.6	57	23.3	61	22.8	49	16.7		56	20.2	33	21.6	44	16.9	65	19.4	
**Tirana**	23	16.9	60	24.5	49	18.4	51	17.3		62	22.4	25	16.3	58	22.3	59	17.6	

^**a**^ Percentage

^**b**^ Mean (SD)

Table 4 reports the associations between gender roles, mobility disability, and SPPB<8. As information on mobility disability was not available for four people, the sample size for this analysis was n = 1963. The unadjusted model shows that, taking androgynous as the reference category, those endorsing the feminine role and those endorsing the undifferentiated roles were more likely to have mobility disability, with PRR = 1.35 (95% CI 1.15;1.58) and PRR = 1.34 (95% CI 1.15;1.56) respectively. These prevalence rate ratios changed very little after adjustment by sex.

**Table 4:** Prevalence ratios (95% confidence interval) for the relationship between gender roles and self-reported mobility disability and poor physical performance, using Poisson regression with robust variance.

Prevalence ratio (95%CI) of self-reported mobility limitations				Prevalence ratio (95%CI) of poor physical performance		
(n = 1963)				(n = 1942)		
Variables	Unadjusted	Adjusted by sex ^a^	Adjusted by all covariates ^b^	Unadjusted	Adjusted by sex ^a^	Adjusted by all covariates ^b^
**Gender roles (ref, androgynous)**						
**Feminine**	1.35 (1.15–1.58)***	1.25 (1.07–1.46)**	1.19 (1.03–1.39)*	1.57 (1.14–2.18)**	1.46 (1.06–2.02)*	1.42 (1.04–1.94)*
**Masculine**	1.07 (0.90–1.28)	1.17 (0.98–1.39)	1.09 (0.93–1.29)	1.08 (0.75–1.56)	1.18 (0.82–1.70)	1.13 (0.79–1.61)
**Undifferentiated**	1.34 (1.15–1.56)***	1.37 (1.19–1.59)***	1.23 (1.07–1.42)**	1.79 (1.33–2.41)***	1.84 (1.37–2.46)***	1.61 (1.20–2.15)**
**Sex (ref, men)**		1.79 (1.58–2.03)***	1.61 (1.41–1.83)***		1.86 (1.46–2.36)***	1.53 (1.19–1.98)***
**Marital status (ref, married)**						
**Single**			1.23 (1.01–1.50)*			1.27 (0.80–2.02)
**Widowed**			1.13 (0.99–1.28)			1.27 (0.96–1.67)
**Divorced**			1.11 (0.92–1.33)			1.31 (0.93–1.83)
**Years of education**			0.97 (0.96–0.99)***			0.96 (0.93–0.99)**
**Income sufficiency (ref, very sufficient)**						
**Barely sufficient**			1.28 (1.07–1.52)**			1.43 (1.00–2.06)
**Insufficient**			1.57 (1.30–1.89)***			1.90 (1.27–2.80)**
**Study site (ref, Kingston)**						
**Saint-Hyacinthe**			0.97 (0.74–1.28)			0.73 (0.44–1.21)
**Tirana**			2.08 (1.63–2.63)***			1.88 (1.21–2.92)**
**Manizales**			1.34 (1.00–1.79)			0.64 (0.35–1.14)
**Natal**			1.30 (0.97–1.75)			1.28 (0.72–2.27)

*p<0.05

**p<0.01

***p<0.001

^a^ Model adjusted for sex only

^b^ Model adjusted for sex, marital status, years of education, income sufficiency, and research site

After adjustment for potential confounders, prevalence rates of mobility disability for participants endorsing undifferentiated roles were higher than for those endorsing androgynous gender roles (adjusted prevalence rate ratio PRR = 1.23, 95% CI 1.07–1.42). Similarly, participants endorsing feminine gender roles had higher prevalence rates of mobility disability compared to those with androgynous roles (adjusted PRR = 1.19, 95% CI 1.03–1.39). Prevalence rates of participants classified as masculine did not differ from those of androgynous participants (adjusted PRR = 1.09, 95% CI 0.93–1.29). To examine whether the associations between gender roles and self-reported mobility varied by sex or across cultural settings, we tested the significance of multiplicative interaction terms. No evidence of interaction between gender roles and sex or research site was detected (p value of multiplicative interaction terms > 0.05). Factors independently associated with mobility disability were being a woman, being single, lower education, insufficient income, and study site. It is noteworthy that controlling for sex did not alter the associations of gender roles and mobility disability. Further adjustment by marital status, education, income and research site slightly decreased the association between gender roles and mobility disability, but did not alter the significance of the results.

Analyses on physical performance were based on 1942 individuals, because 25 subjects were not assessed at the hospital in Manizales. Prevalence rates of poor physical performance for participants endorsing undifferentiated gender roles were higher than for those classified as androgynous (unadjusted PRR = 1.57, CI 1.14–2.18). Similarly, those endorsing feminine gender roles had higher prevalence rates than did androgynous participants (unadjusted PRR = 1.79, CI 1.33–2.41). As with the mobility disability models, prevalence rates for participants classified as masculine did not differ from those for androgynous participants. These prevalence ratios estimates changed little after adjusting by sex and remained strong and significant after adjusting for all potential covariates. There was no evidence of multiplicative interaction by sex or research sites.

As age was not associated with gender roles, it was not included in the multivariate regression models.

## Discussion

### Summary of results

There is abundant evidence showing that women have higher prevalence and incidence of self-reported mobility disability and poor physical performance compared to men. Moreover, the gap between women and men is greater in low and middle income countries, compared with high income countries. However, little is known on whether these sex differences can be explained by gender-stereotyped traits.

In the present study, we were able to test three hypotheses. First, there were significant associations of gender roles with mobility and physical performance of the lower extremities. Second, compared with the androgynous type, feminine or undifferentiated gender roles were positively associated with mobility disability and poor physical performance in older men and women. Contrary to our hypothesis, the endorsement of masculinity traits was not associated with less mobility disability or better physical performance compared to endorsement of the androgynous role. Our findings may be explained by the nature of BSRI-masculinity scale items which mostly capture positive, culturally desirable self-reported masculine or instrumental traits (e.g., leadership abilities, defend owns beliefs). These traits are not limited or conceptually equal to the term hegemonic masculinity that refers to a dominant type of masculinity among a minority of men, which has helped sustain dominance over women [68] and has been linked to a variety of risky or unhealthy behaviors [69]. Third, contrary to our hypothesis, these relationships are not modified by sex or by IMIAS research site.

### Relevance of study findings

The current findings lend support to the androgyny model [28, 70, 71], according to which individuals endorsing androgynous roles are expected to exhibit the best overall lifetime health, while those with undifferentiated roles are expected to show the worst health status. Endorsing androgynous roles offers an advantage of greater behavioral adaptability to the diverse situations a person may experience in daily life.

Previous research demonstrates the persistence of traditional gender roles in populations from Canada, Latin America, and South Eastern Europe, with more women adopting socially desirable instrumental traits due to their active participation in the labor force and their communities [72–77]. Based on our results, we hypothesize that older men and women who are endorsing expressive (feminine) traits or undifferentiated gender roles may not be fulfilling their society’s expectations, may experience loss of mastery over their own lives, may be more likely to adopt poor health behaviors, and have higher risks of chronic diseases and depression. Consequently, they experience more functional limitations.

Our findings suggest that the strength of the associations between gender roles and mobility and physical function change very little after sex adjustment. Thus, gender-related characteristics do not explain sex differences in functional limitations, but are associated independently with them through different pathways. Therefore, future studies should test other hypotheses on the mediation pathways between gender roles and functional limitations.

The IMIAS study provides an opportunity to examine the association between gender roles and functional limitations in five international samples of older adults. Its international design enables us to examine a wide range of exposures and mobility and physical performance outcomes, Moreover, it allows us to compare prevalence rates and identify the causes for differences in population prevalence rates [78, 79]. The countries where our research sites are located differed in gender inequality. Canada ranks 25^th^, Albania 45^th^, Brazil 97^th^ and Colombia 92^nd^ on the Gender Inequality Index published in 2015 [80]. These rankings can be used as approximations of the current national indicators of gender inequality for the participating cities. Thus, Canada is more egalitarian, followed by Albania, and then by Colombia and Brazil, sharing approximately the same position.

Contrary to our hypothesis, heterogeneity of effects across research sites was not observed. In a separate stratified analysis by study site (results not shown), gender roles and both measures of functional limitations of lower extremity function were statistically associated, but wider 95% confidence intervals of the PRR were observed, which is expected due to smaller sample sizes.

### Strengths and limitations

To the best of our knowledge, this is the first study to investigate the potential contribution of gender roles toward understanding the relationship between gender and functional limitations of the lower extremities in a large international sample of older adults. We used measures of gender role and physical function that were validated either in the IMIAS population or in populations similar to those of the IMIAS study [49, 55, 58]. We used both self-reported and objectively assessed measures of physical function of the lower extremities and obtained consistent results.

However, the present study should be considered in light of some limitations. First, as in most cross-sectional studies, we cannot firmly establish the temporal relationship between cause and effect. However, as gender roles are constructed and formed over one’s lifetime, from childhood through adulthood to the early old age period, they probably precede the onset of mobility disability and functional decline.

Second, survival bias may have occurred, since life expectancy at birth in the Latin American populations was very low for the included birth cohorts (it was less than 35 years for the IMIAS cohort of men and women in Natal, Brazil) [81]. The participants in the study may be the hardiest of their cohorts, and may have an overrepresentation of physically fit and androgynous individuals.

Third, as explained in the methods section, the sample at Kingston (Canada) was overeducated compared with the 2006 Canadian census of this city for the same age group, which may limit the validity of our results in Kingston.

Finally, we cannot exclude possible social desirability bias, since the BSRI allows people to rate themselves on various aspects of common cultural values.

## Conclusions

Feminine and undifferentiated gender roles are independent risk factors for functional limitations of the lower extremities in older adults. Our findings suggest that gender roles stemming from the social construction of gender are linked to physical function and mobility in early old age. This study sheds light on how gender roles are associated with mobility disability and poor physical performance in older adults. Further research is needed to examine the mediation pathways through which gender-stereotyped traits influence functional limitations, as well as to investigate the longitudinal nature of these associations.

## Key Messages

### What is already known

- Women have higher prevalence and incidence of functional limitations of the lower extremities than men.
- There are remarkable differences in the magnitude of these differences between men and women across countries.

### What this article adds

- Participants identifying with stereotypical gender role traits defined as ‘feminine’ or ‘undifferentiated’ are more likely to report increased prevalence ratios of self-reported mobility disability and poorer objective assessment of physical performance of lower extremities than ‘androgynous’ participants.
- These associations remained significant after adjusting for sex, marital status, education, income sufficiency, and research sites.

Biological sex and social context are not effect modifiers in this relationship.
